# In-party love spreads more efficiently than out-party hate in online communities

**DOI:** 10.1038/s41598-024-65688-9

**Published:** 2024-07-08

**Authors:** Samuel Martin-Gutierrez, José Manuel Robles Morales, Mariano Torcal, Juan Carlos Losada, Rosa María Benito

**Affiliations:** 1 Complexity Science Hub Vienna, Josefstaedter Str. 39, 1080 Vienna, Austria; 2 Grupo de Sistemas Complejos, ETSIAAB, Universidad Politécnica de Madrid, Av. Puerta de Hierro 2-4, 28040 Madrid, Spain; 3 Department of Applied Sociology, Faculty of Political Sciences and Sociology, Universidad Complutense Madrid, Madrid, Spain; 4 Departamento de Ciencias Políticas y Sociales, Universitat Pompeu Fabra, Trias Fargas 25-27, 08005 Barcelona, Spain

**Keywords:** Twitter, Epistemic bubbles, Partisan echo-chambers, Retweeting, Out-party hostility, In-party affinity, Party supply, Polarization, Complex networks, Scientific data, Human behaviour

## Abstract

In this article, we present the findings of a comprehensive longitudinal social network analysis conducted on Twitter across four consecutive election campaigns in Spain, spanning from 2015 to 2019. Our focus is on the discernible trend of increasing partisan and ideological homogeneity within interpersonal exchanges on this social media platform, alongside high levels of networking efficiency measured through average retweeting. This diachronic study allows us to observe how dynamics of party competition might contribute to perpetuating and strengthening network ideological and partisan homophily, creating ‘epistemic bubbles’ in Twitter, yet showing a greater resistance to transforming them into ‘partisan echo-chambers.’ Specifically, our analysis reveals that the rise of a new radical right-wing party (RRP), Vox, has heightened ideological homogeneity among users across the entire ideological spectrum. However, this process has not been uniform. While users aligned with mainstream political parties consistently share content that reinforces in-party affinity, resulting in highly efficient ‘epistemic bubbles,’ the emergence of the RRP has given rise to a distinct group of users associated with the most extreme partisan positions, characterized by a notable proportion of out-partisan hostility content, which has fostered the creation of low-efficient 'partisan echo-chambers.'

## Introduction

A general tendency among opinion makers, commentators, and pundits is blaming social media for the increasing levels of polarization in contemporary democracies. The logic that underlies this argument is that social media has exacerbated the presence of ideologically homogeneous, like-minded ideological/partisan homophilic networks of citizens^1–3^ with an increasing negative content toward other parties and partisans^4,5^. Evidently, this notion is notable among populist right-wing networks^1,6,7^. However, studies that test these arguments are seemingly contradictory and inconclusive^8–11^. Recent findings demonstrate not only that ideologically heterogeneous exchanges among social media users are deemed more frequent than expected (see^12–16^) but also that ideological/partisan homophily tends to be equally concentrated at both sides of ideological extremes^17,18^. Furthermore, in certain cases, conservative users are more likely to follow media and political accounts classified as left-leaning than the opposite^19^. Additionally, partisan followers tend to react equally to in-party affinity and out-party hostility messages regardless of partisan positioning despite the dominant out-party hostility content in social media^18^.

These inconsistent and conflicting results are based on case studies with limited time frames and non-systematic selection of time periods and centered on the US case^20^. For these reasons, these studies are overlooking the fact that online communication patterns observed among social media users are dynamic, flexible, and situation-specific^1,21^, responding, for instance, to the emergence of a new radical right-wing party (RRP). This tendency leads to the omission of the effect of party competition and changes in party supply, which mainly occur during electoral periods.

In the subsequent sections, this paper presents the results of a social network analysis on four consecutive election campaigns in which we examine the reactions of citizens to partisan content. We use Twitter data, and focus on the retweet mechanism, considered as a proxy of influence. The retweet mechanism serves as a broadcasting tool, implying that users who retweet not only endorse the content of the original tweet but also exhibit a significant level of interest, actively choosing to share the information with their followers^9^. This approach allows us to gauge influence within the Twitter ecosystem, recognizing that the act of retweeting signifies more than a mere acknowledgment—it reflects a deliberate endorsement and interest in propagating the shared content contributing to the creation of ‘informational bubbles’^22^. We argue that those increasingly homogeneous ideological bubbles have been emerging in response to the dynamics of party competition and supply and, more concretely, to the appearance and greater visibility of the challenger parties of both sides of the ideological spectrum. However, with the surfacing of an RRP (Vox), and contrary to the subject of debate among experts and pundits, users with high efficiency (high levels of average retweeting) tend to retweet in-party positive (affinity) messages instead of negative (hostility) ones. At the same time, we note that this change in the right side of the party supply has increased the relative abundance of users that support the most radical challenger parties trying to spread more hostile out-party messages and contributing to the creation of exclusionary ‘echo-chambers’ but with low levels of efficiency (low average retweeting levels). In other words, communication strategies based on promoting out-party hostility seemingly fail to spread out by retweeting even among the most radical followers, failing to create ‘echo-chambers’. Out-party hostility messages not only result in the withdrawal of moderates from social media^23^ but are also, as we will demonstrate, deemed less successful than in-party positive messaging even for parties that try to spread out-partisan hostile messages (see also^24^, pp. 6–7). There is a growing controversy and disagreement on the concept and measurement of “echo chambers”^10^. However, in this article, we adopt the definition provided by Nguyen (^22^, pp. 2–3): "a social epistemic structure in which other relevant voices have been actively discredited… that systematically isolated from all outside epistemic sources from outside epistemic sources… by discrediting outsiders."

This article is based on the results of a longitudinal analysis of Twitter networks and their partisan content in four subsequent national elections in Spain, namely, the 2015, 2016, and May 28 and November 10, 2019, elections. Our analysis employs three metrics for examining the presence of ideologically polarized groups of users as follows: (a) the relative support of each user to the left- or right-wing party block according to their retweeting decisions (a measure of ideological homophily), (b) the impact or communication efficiency (average retweeting) of each user, and (c) the average in- and out-party content spread via retweets. We opted to investigate this phenomenon during electoral campaigns, which are a key moment in which political competition exhibits a visible effect on individual levels of polarization^25^. Additionally, political elites, parties, and candidates tend to behave strategically in their use of and presence on social media at this time^26–29^. The Spanish volatile and changing context constitutes an extremely useful case for testing the research comparative questions on how user reactions generate ideological echo chambers with specific partisan content.

### Theoretical argument

The formation of ideological and partisan informational homophonous communities, stemming from the selection of like-minded discussants, has long been recognized as a significant issue in shaping public opinion^30,31^. However, there is a growing consensus that the advent of social media has intensified this problem, leading to dire consequences in terms of citizen ideological and affective polarization^7,32^ (for a recent controversy about those effects see^9,10,20^). Three major mechanisms can explain this phenomenon. First, the personalized, algorithmically-curated content of social media platforms (i.e., *filter bubbles*) and the increased facility they provide for limiting the exposure to like-minded contents and users^3,33,34^. The second explanation is related to the expressional dimension of political activity via social media that is, sharing, creating, and distributing political content at the click of a mouse, which arguably reinforces the existing beliefs^35^. Both of these processes have facilitated the creation of what have been denominated ‘epistemic bubbles’, which are social epistemic structures formed with no ill intent, through ordinary processes of social selection and community formation^22^. Finally, political parties are increasingly using social media to spread political discourse, which seems to be predominantly negative, discrediting political adversaries^4,5,36^. This trend has led to the formation of ‘echo chambers’ that systematically isolate their members by discrediting ideological and partisan outsiders^22^. These arguments underpin the notion of a growing and avoidable trend towards the existence of partisan/ideologically homogeneous echo chambers with increasing ideological extremism and inter-partisan hostility that contributes to citizens' ideological polarization and the general spread of messages of out-party hostility^2,34^.

However, the preceding literature tends to overlook the importance of party competition and supply and the way users generate and enhance the existing social media networks. This case is especially true during electoral periods^26–29^. Additionally, the reactions of users, which are the product of preexisting attitudes, may condition the effect of party supply communication strategies reinforcing or diminishing the ideological/partisan homogeneity of these informational bubbles. For instance, a number of scholars argue that conservatives would be more likely than progressives to prefer homogeneous informational bubble environments for exchanging opinions due to heightened epistemic, existential, and relational needs for reducing uncertainty, threat, and social discord^2,37–42^. However, certain psychological studies dispute this conclusion and suggest that liberals and conservatives would be equally likely to engage in selective exposure to information and opinion exchange that confirms their preexisting opinions. One study actually found that far-right supporters prefer to be on an ideologically diverse platform like Twitter where they can actively abuse their political opponents^15^. Moreover, conservatives and liberals would exhibit equivalent levels of ideological homophily, insofar as processes, such as dissonance reduction, identity maintenance, and motivated reasoning, which are also highly general and prevalent in the general population^43–48^.

Against this background, we formulate the following research questions (RQs):

- RQ_1_: Are homogeneous ‘epistemic bubbles’ more frequent on one side of the ideological spectrum?
- RQ_2_: Does the arrival of new *challenger parties* to the electoral arena alter the homogeneity of these ‘epistemic bubbles’?

The concept of affective polarization is rooted in social identity theory (SIT)^49,50^ and self-categorization theory (SCT)^51^ which posits that individuals tend to categorize the world into in-groups and out-groups^52^. Based on these two theories people develop some partisan social identities that categorize the world into partisan in-groups (us) and out-groups (them)^53–59^. Recent studies confirm that positive partisanship is a strong driver of individual attitudes and behaviors^60,61^. However, a strong and positive identity with the in-party does not necessarily imply prejudice against the outgroup^62^. Some scholars have even argued that negative partisanship exerts a greater effect on the reactions of citizens^63–66^, regardless of the levels of in-party affinities^53,67^.

This is essential for our discussion about individual behavior in the informational communities in social media. In partisan homogeneous social networks do not need necessarily lead to the promotion of out-party hostility messages that incrementally transform ‘epistemic bubbles’ into efficient ‘echo-chambers’ as currently assumed. The members of those communities might not choose to retweet the negative sentiments and expressions against the other partisans or their parties. In fact, there is evidence that out-party feelings toward other parties tend to be lower among citizens following in-party Twitter accounts^24,68^. Contrary to some existing arguments^5,69,70^, a recent study^18^ presents proof that in-party positive messages seemingly drive predominant reactions.

It might be certain that the presence of negative campaigning in social media is higher among challenger parties^71^. In this respect, Balcells and Padró-Solanet^12^ argue that partisan/ideological homophily tends to increase when the majority of radical right-wing parties (RRPs) or candidates (populist and radical right parties) are dominating major social media networks^23^. This is because these types of parties use social media to attract attention and create a distraction from the mainstream party coverage via emotional and polarizing partisan messages^6,36,72,73^. However, this communication strategy is also common among other types of parties. In general, as mentioned by a few scholars^74–76^, incumbent and challenger candidates of mainstream parties observe the heterogeneous use of social media, which is mainly dependent on the popularity and issue positioning of their candidates. In this manner, the prevalence of these negative messages might or not increase affective polarization through social networking services, but as we discussed above, this does not mean that this type of hostile out-party messages translates higher efficiency (more retweeting) among Twitter users.

Thus, we pose the following RQs:

- RQ_3_: Are out-party hostility messages the most efficient (retweeted) in partisan social media?
- RQ_4_: Is the emergence of extreme challenger parties altering these patterns?
- RQ_5_: Is the majority of the most radical party followers more prone to retweet negative (out-party hostile) or positive (in-party affinity) messages?

### Case study

Spain and, particularly, the period under study (2015–2019) are unique due to their instability and high levels of ideological and affective polarization^77^, which fomented an important level of fragmentation, the emergence of three new challenger parties and the subsequent inability of the country to establish a stable government. The turning point began with the emergence of a new challenger party called *Podemos* (Pod), which was later transformed into *Unidas Podemos* (UP). The party unexpectedly obtained 8% of the vote share and five seats in the European Parliament in the 2014 European election and consolidated its electoral strength in the first election under study, that is, the 2015 general election^78^. *Podemos* was a main actor in the Spanish party system since the 2015 general election (jointly with its regional partner organizations) in which it gained approximately 21% of the vote^79^. At the same time, a second new party, *Ciudadanos* (Cs), also obtained representation in the national parliament for the first time with 14% of the vote share^78^. Cs is a moderate center-right party with a strong stance against the independence of Catalonia, an autonomous region in northeastern Spain with a significant social and political movement advocating for independence. Ciudadanos' anti-Catalan position enabled them to become the most-voted political force in Catalonia in 2017, but their strong anti-Catalan stance has caused them to lean further to the right. Finally, Spain had long been on the exclusive list of countries in Europe without a relevant radical right-wing party.

This situation changed with the sudden rise of Vox, a party that clearly belongs to this radical ideological family^80,81^. Founded in December 2013, Vox first gained political representation in the regional election held in December 2018 in Andalusia, the most populous Spanish region in which it obtained 10.96% of the regional vote and 12 seats (out of 109). This regional election propelled Vox nationwide and placed it at the center of the electoral campaign for the Spanish national election held in April 2019 in which the party obtained 10.26% of the vote and 24 seats (out of 350). Although Vox obtained more modest results in the subsequent local, regional, and European Parliament elections held in May 2019, the party significantly increased its representation in the November 2019 repeated national election (15.98% of the vote and 52 seats). Despite the new interparty dynamics and disputes, this transformation resulted in an inter-bloc ideological confrontation between two major groups at the national level^82,83^, namely, the left (formed by UP and the socialist *Partido Socialista Obrero Español* [PSOE]), and the right (formed by Cs, the conservative Partido Popular [PP], and Vox).

The surprising irruption of Vox into the Andalusian election at the end of 2018 produced important consequences for the subsequent electoral campaign ahead of the 2019 April national election. On the right side of the political spectrum, PP and Cs rejected a *cordon sanitaire* (a political strategy employed by mainstream political parties to isolate or exclude RRPs from political power or influence) against Vox and accepted external support from that radical right-wing party for the formation of a joint PP–Cs regional cabinet in Andalusia. Moreover, they did not deny the possibility that the same formula could be repeated at the national level if the results made this coalition feasible. Eventually, the media began to call PP, Cs, and the Vox the “right-wing tripartite.” On the left side of the spectrum, PSOE and UP mobilized their electorate against the threat posed by a potential right-wing government with the participation of the radical right^84^.

This period under study was also one of great government instability. Since the successful motion of no confidence, which resulted in the downfall of the conservative government of Mariano Rajoy and the establishment of Socialist Pedro Sánchez as the new Prime Minister in June 2018, center-right parties initiated a harsh opposition to the Socialist government. Notably, the leader of the PP at that time, Pablo Casado, went as far as to accuse Sánchez of being an “illegitimate” Prime Minister. Hence, a reasonable expectation is that the moderate-right partisans, which held the highest levels of animosity toward left-wing supporters, were the ones who switched their support to the emerging Vox. In addition, in the years before the emergence of Vox, a portion of the Spanish electorate on the right and center lacked a clear partisan preference. In other words, the PP was in disrepute as a result of successive corruption scandals and its controversial handling of the Catalan crisis, and the programmatic and strategic erratic fluctuations of the Cs were reflected in a volatile voting intention in the polls on the right side of the political spectrum^85^.

## Data and methods

The main source of data is the social networking site Twitter. The concise character of a tweet (a message posted on Twitter) makes the platform a powerful tool for sharing breaking news and participating in dynamic debates. It is one of the most used media by journalists, politicians, and political enthusiasts for sharing views and news^86^. Moreover, Twitter’s Application Programming Interface (API) enabled researchers to retrieve user data, providing access to a portion of the digital footprint of users with relevant information for the examination of multiple phenomena, including influence, sentiment, and polarization, among others. In the last quarter of 2015, this social network recorded 305 million active users per month, which increased to approximately 330 million in 2019^87^.

We used the Twitter streaming API to download tweets that match a set of keywords in real time. To minimize partisan bias, we selected a set of neutral keywords for filtering messages. We do so by including nonpartisan hashtags related to the election dates and, for the 2019 elections, also a balanced set of partisan keywords, so that we have a comparable number and type of keywords associated with each relevant party (names, main candidates, slogans, etc.).Table 1 presents the keywords used to build each dataset and the number of users that participated in each conversation, the number of tweets they published, and the time period considered for each case.

**Table 1:** Description of Twitter datasets.

Dataset	Keywords	No. of tweets	No. of users	Time interval	Time span/days
Spanish elections 2015	20D, 20D2015, #EleccionesGenerales2015	1,796,093	409,410	2015/12/04–2015/12/21	18
Spanish elections 2016	26J, 26J2016, #EleccionesGenerales2016, #Elecciones26J	2,814,781	174,110	2016/06/10–2016/06/27	16
2019 28A Spanish elections*	https://pastebin.pl/view/84efeb1b	17,706,517	1,339,514	2019/04/11–2019/04/29	19
2019 10N Spanish elections*	https://pastebin.pl/view/84efeb1b	18,887,413	1,975,843	2019/11/01–2019/11/11	11

*We deposited the keywords in an online repository due to the high quantity used for the 2019 elections in Spain.

Our analysis is focused on one of the most common forms of interaction on Twitter, retweeting, which involves reposting the content of another user with an attribution to the original author. Retweeting can be viewed as an indicator of information diffusion and message endorsement^88^. We utilized three metrics to examine the presence of ideologically polarized echo chambers and their partisan content. The first is an opinion ideological index associated with each user and ranges between − 1 and + 1, which captures the estimated support of different users for left- (− 1) and right-wing (+ 1) party blocks. The second denotes the impact capacity or communication efficiency of each user, which is measured as the average number of retweets obtained for each posted tweet and constitutes an excellent proxy for its impact among Twitter users. The last one is the level of in-party affinity and out-party aversion of the content that was spread via tweets and retweets. We combined these three pieces of information to address the abovementioned central questions.

To compute the opinion index ${x}_{i}$ of each user $i$, we first identify user accounts associated with politicians and partisan institutions and classify users belonging to UP and PSOE under the left-wing block and those from PP, Cs, and the Vox under the right-wing block. We then expand this initial set of partisan Twitter accounts by pinpointing popular and engaged users found in the same clusters of a retweet network. We use this set of users as *opinion seeds* with an opinion index of − 1 (left-wing block) or + 1 (right-wing block). We infer the opinions of the rest of the users by spreading these opinion values throughout the retweet network, such that each user’s opinion is the average of its neighbors’ opinions. In other words, the opinions of users who retweet opinion seeds are influenced by those seeds, and the opinions of users who retweet users who retweet opinion seeds are also influenced, and so on. For example, a user retweeting only politicians from the left-wing block will be assigned an opinion of − 1; however, if the user retweets politicians from both blocks at the same rate, then the user will be assigned an opinion of 0 (neutral). In summary, this method intends to map out the opinions of Twitter users based on their engagement with political content and their relationships with other users on the platform. Users that are close in the retweet network will have similar opinion indices, so a concentration of users with very similar opinion indices may correspond to a clustered region of the network with tight connections. Other scholars use a relatively similar strategy for measuring the level of ideological homophily (echo chambers) of Twitter networks^1^. Sections 1A and 2A of the of the Appendix provide the technical details, including the computation of the opinions of users that do not directly retweet any opinion seed.

Information diffusion is one of the core functions of Twitter. Most users publish messages that target not only their immediate followers but also a wider audience through retweets. Retweet counts can then be used to estimate the ability of a user to propagate information, but this simple metric does not consider the effort required for the user to obtain these retweets. A user that gets 100 retweets after publishing two messages is more influential than one that achieves 100 retweets after publishing 200 messages. Therefore, we calculate the ratio of the number of retweets obtained by a user (${r}_{i}$) by the number of tweets published (${a}_{i}$) to obtain a measure of user efficiency or impact capacity^89,90^ using Eq. (1):

1 $${\upeta }_{i}=\frac{{r}_{i}}{{a}_{i}}$$

This metric quantifies the relationship between the individual activity of the user and the spontaneous collective reaction triggered by the user among peers. In the abovementioned example, the popular user that attains 100 retweets for two tweets has $\upeta =50$, while the one that obtains 100 retweets for 200 tweets has $\upeta =0.5$.

**Figure 1:**
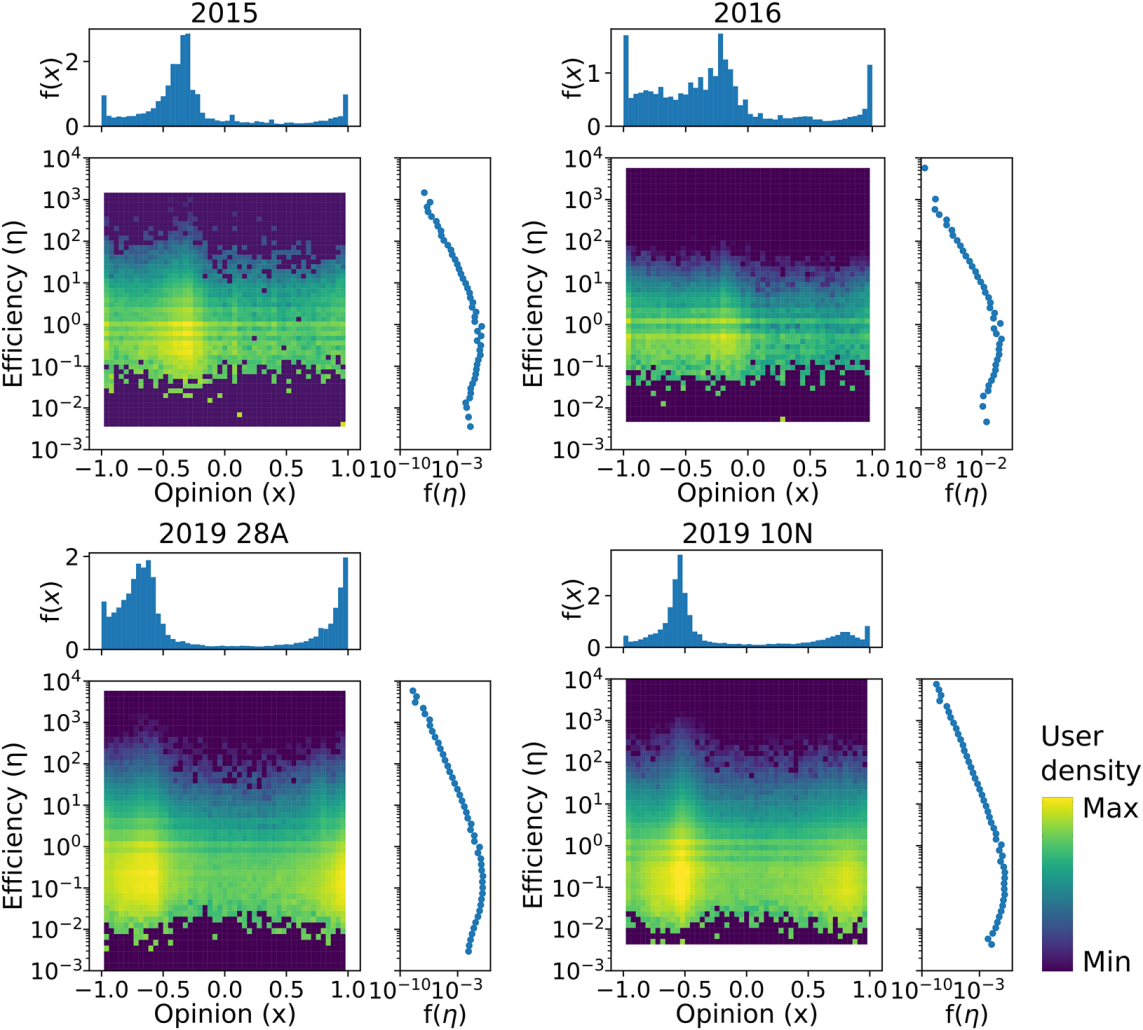
Opinion-efficiency maps. Heatmaps showing the empirical joint probability distributions of opinion and efficiency ${\text{P}}_{\text{emp}}(\text{x},\upeta )$ for the four electoral contexts. The marginal distributions of opinion and efficiency are respectively shown at the top and right side of each plot.

Once we have computed the opinion index ${x}_{i}$ and efficiency value ${\upeta }_{i}$ of each user, we calculate the joint probability distribution ${P}_{emp}\left(x,\upeta \right)$, which tells us the proportion of users with a given opinion and efficiency; that is, the fraction of $\left({x}_{i},{\upeta }_{i}\right)$ pairs. We computed the empirical joint probability distributions ${P}_{emp}\left(x,\upeta \right)$ by building 2D histograms. We represent them as heatmaps in Fig. 1. The x-axis encodes the left–right ideological spectrum built using the opinion index. The y-axis represents the efficiency $\upeta$ in logarithmic scale, which ranges from 0.001 to 1000 retweets per tweet. For example, a left-leaning user that obtains 20 retweets on average for each of her tweets would be placed in the upper left quadrant of this map. Regions with lighter colors have a higher density of users. The marginal distributions of opinion and efficiency are shown as 1D histograms at the top and right sides of each plot. The y-axis of the plots ($\upeta$) is shown in logarithmic scale to facilitate the visualization of the results because the efficiency is a highly heterogeneous magnitude in Twitter conversations^89,90^.

We compared the empirical ${P}_{emp}\left(x,\upeta \right)$ distribution to the one that would be obtained if opinion and efficiency were independent random variables; that is, if there were no actual relationship between them. In that case, the joint probability distribution would be the product of the marginal distributions: ${P}_{null}=P\left(x\right)P\left(\upeta \right)$. Comparing this null distribution to the empirical one, we obtain an opinion-efficiency map of relative user density:

2 $${P}_{diff}=\frac{{P}_{emp}-{P}_{null}}{{P}_{null}}$$

This measure of relative user density helps us identify areas of interest such as those where there are more users than expected (${P}_{diff}>0$) or fewer users than expected (${P}_{diff}<0$). For each electoral context, we computed the relative user density according to Eq. (2). To do so, we first obtained the null joint probability distribution for the case where $x$ and $\upeta$ are independent.

**Figure 2:**
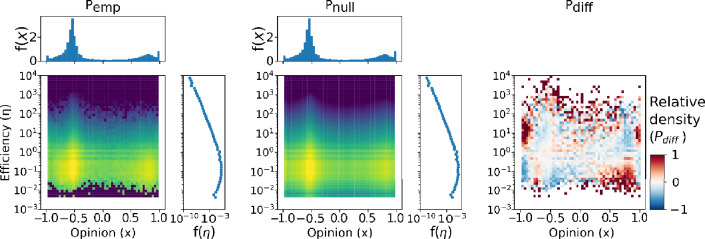
Building the map of relative user density. Comparison between the empirical joint probability distribution (${P}_{emp}$), the independent variables null distribution (${P}_{null}$), and their normalized difference (${P}_{diff}$), computed according to Eq. (2).

**Figure 3:**
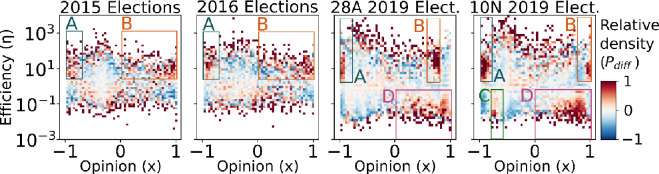
Opinion-efficiency anomaly maps. Each heatmap shows the relative overabundance (red) or underabundance (blue) of users with particular values of opinion and efficiency.

As an example of this process, Fig. 2 illustrates the empirical distribution of the 2019 10N elections, the null distribution, and the resulting map of relative user density. Notice that, by construction, the null distribution has the same marginals as the empirical one, so the histograms at the top and right sides are the same in both panels. Crucially, the strongest anomalies (darkest colors of the rightmost panel of Fig. 2) do not necessarily coincide with the areas of high absolute user density, revealing non-trivial behavioral patterns. In Fig. 3, we show the opinion-efficiency anomaly maps for the four electoral contexts. The red areas of the maps indicate regions of overabundance of users given the opinion and efficiency distributions. We focus our analyses on the regions of high relative user density marked A–D on the plots. The anomaly maps in the 2015 and 2016 elections are noisier due to the smaller user sample, and therefore more prone to show superfluous fluctuations. But as we will show below, the tweet contents in those areas are remarkably consistent across elections, indicating that the user sample is big enough to be representative.

**Figure 4:**
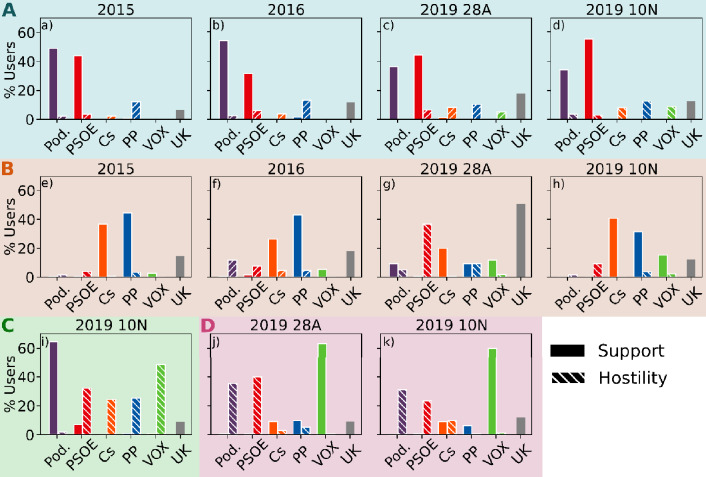
In-party support and out-party hostility. Support and opposition to the 5 main political parties by the users in the areas A-D marked in Fig. 3. The label “UK” corresponds to “unknown.” Each user can be assigned more than one label; for example, by supporting one party and criticizing another. Thus, the bars may sum up to more than 100%.

We then use the third metric to examine the party content of these selected anomalous areas in the opinion-efficiency maps. To analyze their respective party content, we extract a random sample of users of each area and manually annotate their tweets as supportive or critical of the parties. The annotators were instructed to label a user as supportive of a party only if their tweets contained explicit mentions to the party or its main members and there were words or expressions clearly indicating support for that party; analogous criteria were followed for labeling users showing hostility towards a party. Section 3A of the Appendix provides all the details about the user selection and annotation criteria. Finally, we count the proportion of sampled users that exhibit in-party affinity or out-party hostility. Figure 4 depicts these quantities. For example, in area A of the 2015 elections, which spans between − 1 and − 0.7 in the opinion axis and between 2 and 1000 retweets/tweet in the $\upeta$ axis (Fig. 3, left), approximately 45% of the users displayed support for Podemos (Fig. 4a).

## Results

As shown in Fig. 3, we first detected areas A and B of high relative user density characterized by high information dissemination efficiency, which is seemingly reproduced in the four consecutive elections. We found that area A is located on the left position from the very beginning of the electoral period. This area presents two important characteristics. First, it is an ideologically homogeneous, leftist ‘epistemic (communication) bubble’; second, it is characterized by the dominance of positive in-party messages in favor of *Podemos*, the extreme-left populist party that emerged in 2014, and the Socialist party -PSOE- (see panels a–d of Fig. 4). Thus, although we do not have data prior to 2014 to support the argument, a possible interpretation is that the presence of this leftist highly homophilic communication bubble could have been fostered by the emergence of *Podemos* months prior to this election. Nevertheless, this area of high relative user abundance and efficiency remains stable across the four consecutive elections.

Area B is also an area of high relative user density with high efficiency, but it displays different characteristics and patterns of stability over time compared with those of area A. During the first two elections, the ones celebrated in 2015 and 2016, this area was not a right-wing ideologically homogeneous communication bubble, as indicated by the wide range of opinion values with overabundance of users. Additionally, the dominant presence of positive in-party messages toward Cs, an ideology-centered party, and PP, a right-wing party, characterized this area (see panels e and f in Fig. 4). However, the level of homogeneity of this area changed with the arrival of Vox at the national arena at the end of 2018 and at the beginning of 2019, which was immediately after the national election on May 28, 2019. The emergence and increasing success of this RRP deteriorated the level of support of the PP, which disputes its predominant position on the right-side ideological spectrum and renders the two parties competing for the representation of the *real right*. This scenario also affected Cs, which began to adopt more belligerent conservative strategies and, thus, abandoned the more centrist ones^91^.

All of these changes in the party supply are also reflected in two significant changes in area B. First, this area evolved from being a heterogeneous area of right-wing partisan exchanges to a more homogeneous ideological informational bubble, spanning a thinner interval of the opinion axis in the last two electoral periods, as observed in Fig. 3. This finding confirms those of other studies that demonstrated that inter-ideological exchanges in Spain over Twitter tend to decrease as extremists gain protagonism in these networks^12,92^. Second, this area of high efficiency was characterized by the dominant presence of negative messages toward the PSOE and a certain significant increase in in-party positive messages toward this new extreme right-wing party (Fig. 4g). These changes illustrate the adaptation of Twitter users to the changing dynamics of party competition, transforming this ‘epistemic bubble’ into a right-wing ideological exclusionary  ‘echo-chamber’ for those particular elections.

The adaptation of these areas of high efficiency (average retweeting) of party competition continues the following election on November 20, 2019. These elections were called due to the failure of Sánchez, the leader of the most voted party in the preceding elections (PSOE) to obtain a sufficient majority to form a government with the opposition of a right-wing front composed of Cs, PP, and Vox. These parties were adopting a unified increasing confrontational conservative discourse. In line with this party competition dynamics, area B reflected the reactions of users evolving to the important presence of in-party positive messages toward Vox, PP, and Cs but with a significant decrease in the levels of party hostility toward the PSOE. The out-party hostility present in area B in the preceding elections of May of the same year (Fig. 4g) was seemingly transformed within a few months into an area dominated by in-party positive affinity toward the three parties of this side of the ideological spectrum (Fig. 4h). This change in area B indicates that this high retweeting-efficient areas tend to be transformed from areas of high hostility (‘echo-chambers’) to more in-party affinity ones (‘epistemic bubbles’) even if they contain certain radical partisan voters.

We confirmed this predominance of cross-party efficient reactions by analyzing the emergence of other areas of high relative user density that present an important *anomaly*, namely, areas C and D, whose party content is displayed in panels i–k of Fig. 4. These areas with overabundance of users present particularly low levels of retweeting. Area D on the ideological right, which was detected for the first 2019 elections (two panels on the right of Fig. 3) and repeated a few months later (November 10 elections) is characterized by high levels of out-party hostility toward *Podemos* and PSOE with a significant number of in-party affinity messages toward Vox (Fig. 4j–k). The presence and content of this area illustrates that out-party hostility diminishes retweeting levels even among the most radical partisan users that display high levels of in-party affinity toward this RRP.

Area C, with high user overabundance and increased ideological homogeneity (thin range of the opinion axis) but low levels of efficiency (see the last right side panels of Fig. 3), seemingly emerges from the left side of the ideological spectrum most likely as a reaction of the successful emergence of Vox in the political area in the preceding electoral elections of April 2019, just a few months before. This new emerging area is also characterized by high levels of in-party messages (toward *Podemos*) but high levels of anti-party messages toward other parties, naturally mostly directed towards that particular RRP (Fig. 4i). These results confirm that ideologically homophilic informational communities (‘epistemic bubbles’) on Twitter are only seemingly effective in areas that contain positive in-party messages. Highly efficient areas only emerge when out-party hostility is absent. When Twitter messages are dominated by in-party messages toward the most extreme parties combined with out-party hostility, the majority of users opt to not retweet. Therefore, contrary to the usual assumption, the majority of Twitter users are reluctant to contribute to the diffusion of negative messages toward the other parties even among the most radical party users/supporters, resisting the temptation to be transformed into exclusionary/negative partisan ‘echo-chambers’.

## Discussion

In the preceding longitudinal study on Twitter users during the electoral campaign of four Spanish elections, we demonstrated how retweeting has incrementally resulted in ideologically homogeneous ’epistemic bubbles’ responding to the dynamics of party competition and the emergence of challenger parties at the extreme side of the ideological spectrum. We also depicted that these areas are efficient (high levels of average retweeting) if they contain in-party affinity messages. ‘Echo-chambers’ characterized by the high presence of hostile messages from parties seemingly result in low levels of retweeting, even among extremist party supporters.

In summary, this study aims to contribute to two empirical and theoretical arguments. First, it illustrates that the generation of more ideologically homogeneous informational bubbles is dependent on party competition and changes in-party supply, and more concretely to the emergence of challenger parties regardless of their ideological position (see the effect of the emergence of Podemos -leftist- and Vox -RRP- in the ideological concentration of the informational bubbles after their emergence in 2014 and 2018 respectively). Second, contrary to a few studies, we found that in-party affinity messages are the most efficient (most frequently retweeted). In fact, messages containing hostile out-party messages tend to obtain few retweets even among the most radical party followers.

These findings align well with previous studies^9,93–95^ that also consider retweets as a reliable proxy for influence. Retweets are regarded as a broadcasting mechanism, indicating that the user engaging in the retweet not only agrees with the original tweet but actively supports and endorses its content. A notable example is the Spanish general election of 2011, where research demonstrated a high assortativity coefficient over the matrix defining the fraction of edges going from one party to another. This high coefficient suggests that politicians primarily retweeted individuals from their own party^93^. Similar observations were made during the 2010 U.S. Congress elections, revealing that retweets were more ideologically polarizing than mentions among regular users^96^. These instances collectively highlight the role of retweets as a potent indicator of influence, capturing not only agreement but also active endorsement and support within the online political discourse.

Additionally, we infer that citizens are learning about social media over time and interact with it according to the specific partisan content they intend to spread^23^. In this manner, social media users are increasingly producing two types of user profiles. The first is formed by citizens that mainly use ideologically homogeneous communication bubbles to express in-party affinity. The second, emerging as a consequence of the successful electoral emergence of RRPs, is characterized by users supporting new challenging parties that intend to express out-party hostility content resulting in the creation of ‘echo-chambers’ but obtaining very low efficiency. This has implications for the communication strategies that may be adopted by party elites since it confirms the negative implications of politicians who predominantly use negative content in their Twitter messages^4^. If tweets or retweets produced or reproduced by partisan Twitter users express more in-party affinity, then politicians would be encouraged to adopt this strategy, which reduces the presence of a toxic political climate fed by out-party hostility^97^.

However, this study has its limitations. First, although the evidence clearly indicates that in-party positivity is seemingly the most efficient communication content on Twitter, we need to point out that we cannot disentangle that the low efficiency in the most radical areas might be due to either the dominance of in-party messages in favor of the most extreme radical parties or to the significant presence of out-party hostility messages. Second, we approximated the complex ideological space of a multiparty democracy using a left-/right-wing axis, which grouped moderate and extremist parties together. Although this simplification is widely used, multiparty democracies are typically multidimensional, such that additional axes (e.g., perceived extremism) are required to fully characterize this ideological space and enable the distinction between parties of a similar ideology^98^.

### Supplementary Information

Supplementary Information.

## Data Availability

The data that support the findings of this study are available from the corresponding author upon reasonable request.
