# Supplementary material for: In-party love spreads more efficiently than out-party hate in online communities
Source: Sci Rep. 2024 Jul 8;14:15700. doi: 10.1038/s41598-024-65688-9 (PMC11231175; doi:10.1038/s41598-024-65688-9)
Supplement: Supplementary file 1 — Supplementary Information. [file 41598_2024_65688_MOESM1_ESM.docx]

**Appendix to:**

**In-party love spreads more efficiently than out-party hate in online communities**

**Samuel Martín Gutiérrez**

**Complexity Science Hub Vienna**

**Josefstaedter Str. 39, Vienna 1080, Austria**

**Grupo de Sistemas Complejos**

**ETSIAAB, Universidad Politécnica de Madrid**

**Av. Puerta de Hierro 2-4, Madrid, 28040, Spain**

[**martin.gutierrez@csh.ac.at**](mailto:martin.gutierrez@csh.ac.at)

**José Manuel Robles Morales**

**Department of Applied Sociology**

**Faculty of Political Sciences and Sociology**

**Universidad Complutense Madrid**

**Madrid, Spain**

**Universidad Complutense de Madrid**

[**jmrobles@ucm.es**](mailto:jmrobles@ucm.es)

**ORCID: 0000-0003-1092-3864**

**Mariano Torcal**

**Departamento de Ciencias Políticas y Sociales**

**Universitat Pompeu Fabra**

**Trias Fargas 25-27, Barcelona 08005, Spain**

[**mariano.torcal@upf.edu**](mailto:mariano.torcal@upf.edu)

**ORCID: 0000-0002-0060-1522**

**Juan Carlos Losada**

**Grupo de Sistemas Complejos**

**ETSIAAB, Universidad Politécnica de Madrid**

**Av. Puerta de Hierro 2-4, Madrid, 28040, Spain**[**juancarlos.losada@upm.es**](mailto:juancarlos.losada@upm.es)

**ORCID: 0000-0002-4373-603X**

**And**

**Rosa María Benito**

**Grupo de Sistemas Complejos**

**ETSIAAB, Universidad Politécnica de Madrid**

**Av. Puerta de Hierro 2-4, Madrid, 28040, Spain**

[**rosamaria.benito@upm.es**](mailto:rosamaria.benito@upm.es)

**ORCID: 0000-0003-3949-8232**

Corresponding autor: **Samuel Martín Gutiérrez**

martin.gutierrez.samuel@gmail.com

*1A. Opinion inference*

Our opinion inference technique is based on the interaction network built from users' retweets. Retweets are a broadcasting mechanism that usually imply that the retweeting user agrees with the original tweet, so they can be used as a proxy of influence [1], [2], [3]. We build the retweet network by considering each user as a node and making a directed link from user *i* to user *j* when *i* retweets *j*. Then, we identify the opinion leaders of the network and their respective ideologies. Since we are studying electoral contexts, we choose the opinion leaders from the engaged and influential users associated to the different political parties, so their opinions are determined by their parties' ideological leaning.

In the 2015 and 2016 elections, the main right-wing parties were Ciudadanos (Cs) and Partido Popular (PP), and the main left-wing parties were Podemos and Partido Socialista Obrero Español (PSOE). In the 2019 elections, we included Vox as an additional right-wing party. We use this small subset of leaders (the *elite*) as opinion seeds. To infer the opinions of the remaining users (the *listeners*), we use a learning model based on the DeGroot's process [4], [5], [6] that propagates the elite's opinions throughout the network [7]. In this model, each *elite* node is assigned a fixed opinion. Then, the opinions of the *listeners* are computed as the average of the opinions of their neighbors.

More formally, let us assume that we have $N$ nodes labeled as $i=1,2,\ldots,N$ with opinions $x_{i}\in\left[ -1,+1 \right]$. The opinion of an elite node is fixed to either $-1$ if she leans left or $+1$ if she leans right. We compute the opinions of the listeners iteratively by computing a weighted average of their neighbor's opinions:

$$x_{i}\left( t \right) = \frac{\sum_{j} A_{ij} x_{j}\left( t-1 \right)}{\sum_{j} A_{ij}}$$

( 2 )

Where $x_{i}\left( t \right)$ is the opinion of user $i$ in time step $t$ and $A_{ij}$ is the number of times user $i$ has retweeted user $j$. For elite nodes, $x\left( t=0 \right)=x\left( t=1 \right)=\ldots=x\left( t=\infty\right)=-1 or +1$, because they are the opinion seeds, and their opinions are constant throughout the process. Listener nodes are assigned an initial neutral opinion $x(t=0)=0$. Then, Eq. (2) is applied asynchronously to random nodes until their opinions have converged. The convergence criterion is $\sum_{i=1}^{N} \left| x_{i}\left( t \right)-x_{i}\left( t-N \right) \right|<{10}^{-6}$. That is, the sum of all the absolute opinion differences between time $t-N$ and a time $t$ after $N$ nodes have updated their opinions must be $\approx0$.

*2A. Selection of the elite*

Elite nodes are crucial because they are the opinion seeds that are used to infer the ideology of the remaining nodes. Since we are studying Twitter data, the elite is chosen by analyzing behavioral patterns of the users (which are the nodes). The selection is carried out in two steps.

First, we look for highly engaged and influential users with well-defined opinions. The engagement of a user is quantified by the proportion of days (out of the total duration of the period under study) that the user participates in the conversation. We call this magnitude the *participation ratio*. The influence of a user is measured by the number of retweets received. To get a reasonable number of engaged and influential users, the specific thresholds for participation ratio and retweets are adjusted for each dataset. Nevertheless, previous studies have shown that the resulting opinion distribution is robust with respect to fairly large changes in the thresholds [8], [9].

Once these engaged and influential users have been selected, in the second step we check which of them can be assigned to an opinion pole (in this study, politicians and supporters of left-wing or right-wing parties). We do that by computing the communities of the whole retweet network with the Nested Stochastic Block Model [10]. This model is hierarchical, which means that it reveals the community structure of the network at different levels. The first level corresponds to the individual nodes, and the higher the level, the lower the number of communities it has. Several communities of a given level may merge into a larger community in a higher level. The highest level has only one community that corresponds to the whole network.

We calculate the community structure of the retweet network with the aforementioned model and analyze the community assignment of the elite users that we selected in the first step. Then, we look for the highest level of the community structure where each community contain nodes only from one faction (or from none, as some contain users with no clear support for any pole, such as regular users, institutions, journalists...). If there are nodes from two different poles, we go to an inferior level of the community structure. We use contextual information to identify the factions within the community structure. In particular, we look for elite nodes corresponding to user accounts clearly associated to a political party (the party leaders, their institutional accounts, etc.). The process can be considered as a *snowball* sampling technique, as the user subset of each pole is built from a small sample of nodes with known affiliation. Once the communities where we can find users with a clear affiliation to a pole are identified, we assign all the users inside those communities to the same pole. To avoid wrongly including potentially neutral nodes in the elite, we have elaborated a list of media accounts (TV, radio, press...) which are removed from every elite set. Even if many media have a clear ideological leaning, we prefer not to fix their opinions a priori and assign them one with the inference technique to minimize biases.

Once the elite nodes have been selected, we filter out all the users that have no directed path starting on them and ending in an elite node, as their opinions cannot be inferred from their connections. In other words, we require every listener to have retweeted someone who has retweeted someone who, after a succession of retweet connections has retweeted an elite user. We call this sub-network the elite-connected network (Atienza-Barthelemy 2019).

In Table 1A we present the minimum number of retweets ($RT_{min}$) and minimum participation ratio ($PR_{min}$) considered to filter influential and engaged users, as well as the number of users obtained by applying this filter and the number of users finally selected as elite after analyzing the community assignment of the filtered users. The number of listeners and links of the corresponding retweet network is also shown.

**Table 1A. Minimum retweets (**$\boldsymbol{R}\boldsymbol{T}_{\boldsymbol{min}}$**) and minimum participation ration (**$\boldsymbol{P}\boldsymbol{R}_{\boldsymbol{min}}$**) used to filter the influential and engaged users (Initial Elite) for each Twitter conversation**.

| Elections | $RT_{min}$ (Tweets) | $PR_{min}$ (%) | Initial Elite | Selected Elite | Listeners | Links |
| --- | --- | --- | --- | --- | --- | --- |
| 2015 | 100 | 50 | 967 | 342 | 236855 | 730802 |
| 2016 | 100 | 50 | 1088 | 560 | 218643 | 699585 |
| 2019 28A | 1000 | 70 | 1485 | 631 | 837056 | 7148478 |
| 2019 10N | 800 | 58 | 1996 | 373 | 957873 | 8197547 |

Note: The Selected Elite corresponds to the number of users of the Initial Elite that have been assigned to a pole considering the community structure and contextual information. The number of Listeners and the number of Links of the final elite-connected network are also shown.

In Figures 1A through 4A we show the hierarchical community structure of the influential and engaged users of each system according to the block model. In this hierarchical structure, the first level corresponds to the individual nodes; in the second level, nodes are aggregated in small communities, and the upper levels progressively merge the nodes into larger and larger communities until the top level is reached, where the whole network is in one community. In each figure, the nodes of the outermost circle are the influential and engaged users. The nodes are grouped and colored according to the communities of the lowest level shown. Those same communities are represented as black squares in the second outermost circle. Then, in each inner circle some of the communities of the immediate lower (outer) level merge until all of them are in the same community at the top level. We have labeled the levels such that level 0 corresponds to the one with smallest communities (the level immediately above individual nodes) and the level with highest number corresponds to the full network.

In Figure 1A we show levels 2 to 5 of the Spanish elections of 2015. In this context, we find *monopolar communities* with clear associations to the different parties at level 2 of the hierarchy. As can be appreciated in the figure, there are two communities belonging to Podemos that merge in the immediate upper level, but the two communities of PP do not merge until the top level. This counter-intuitive phenomenon may arise for two reasons. One reason is related to the properties of the system, as the nodes inside each of the two PP communities belong to different *sections* of the party: in one of the communities we find the main user accounts (the official account of the party, the party's candidate, etc.), while in the other one we mainly find accounts associated to politicians and party branches from peripheral regions of Spain (Andalusia, Galicia, and Catalonia, for example). The other plausible reason stems from the criterion of the adopted block model to assign nodes to one community or another. While many community detection algorithms aim to find groups of nodes with dense intra-connectivity between them and sparse inter-connectivity to the rest of the network, the block model considers all possible connection patterns when grouping nodes in a community. For example, in a star-like network it may place all periphery nodes in one community and all the core nodes in another, as those two blocks provide a very good summary of the network topology. Therefore, the separation into two communities may also emerge due to the different communication patterns of the users inside them. In any case, notice that a thick bundle of links joins the two communities. The number of elite users associated to each pole are presented in Table 2A.

**Table 2A. Number of elite users assigned to each party of the Spanish elections of 2015, 2016, 2019 28A, and 2019 10N.**

| Party | 2015 | | 2016 | | 2019 28A | | 2019 10N | |
| --- | --- | --- | --- | --- | --- | --- | --- | --- |
|  | by party | by pole | by party | by pole | by party | by pole | by party | by pole |
| Vox | - | 191 | - | 235 | 210 | 380 | 89 | 188 |
| PP | 89 |  | 139 |  | 79 |  | 33 |  |
| Cs | 102 |  | 96 |  | 91 |  | 66 |  |
| PSOE | 49 | 151 | 122 | 325 | 77 | 251 | 83 | 185 |
| Podemos | 102 |  | 203 |  | 174 |  | 102 |  |

In Figure 2A we show levels 2 to 7 of the Spanish elections of 2016. Again, we choose level 2 to select the elite and we see that Podemos and PP are assigned two different communities each. The communities do not merge until reaching level 5, like most of the communities of the network. This may be explained with the same arguments presented above: either they correspond to different branches of the parties or present differential communication patterns that cause the model to put them in different groups. The number of elite users associated to each pole are also presented in Table 2A.

In Figure 3A we show levels 2 to 7 of the 28A elections of 2019 with the corresponding communities labeled according to the parties they have been assigned to. The number of elite users associated to each pole are presented in Table 2A.

In the 10N elections of 2019, whose community structure is presented in Figure 4A, we need to choose an even lower level (level 1) to find appropriate communities and, consequently, each pole is assigned a greater number of communities to obtain enough elite users. The number of elite users associated to each pole are presented in Table 2A.

**Figure 1A. Levels 2 to 5 of the hierarchical community structure for influential and engaged users of the 2015 elections system.**


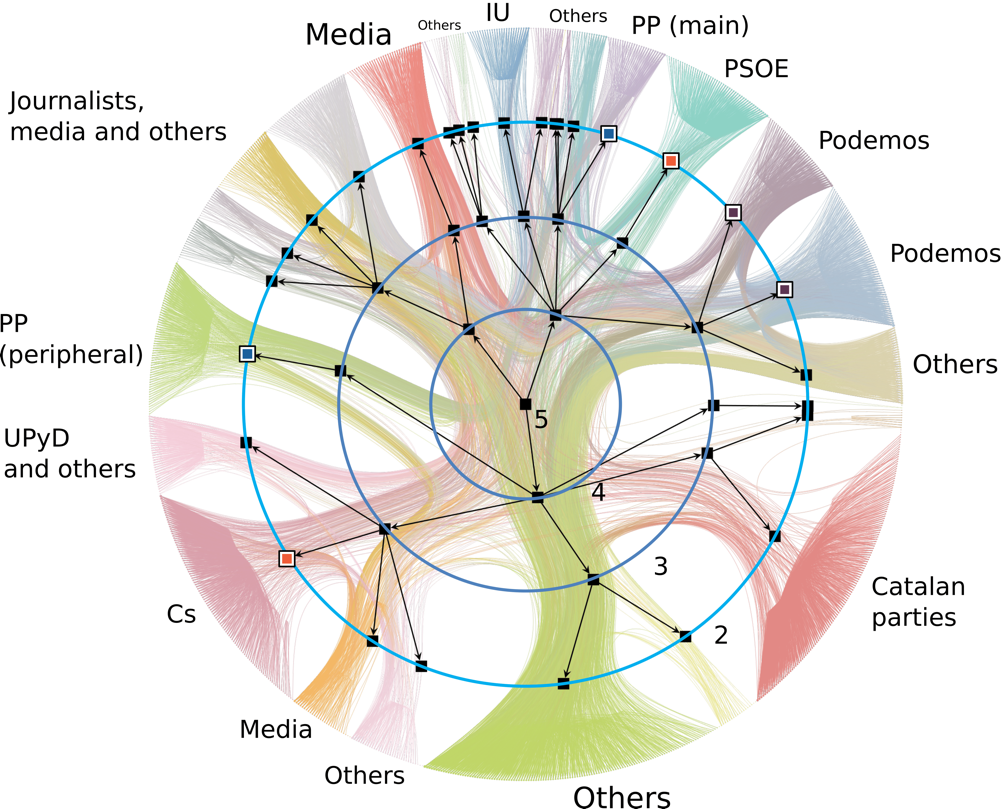


Note: The level used to build the elite is highlighted in light blue and the specific communities assigned to each pole are marked with squares of different colors. Blue: PP. Red: PSOE. Purple: Podemos. Orange: Cs.

**Figure 2A. Levels 2 to 7 of the hierarchical community structure for influential and engaged users of the 2016 elections system.**


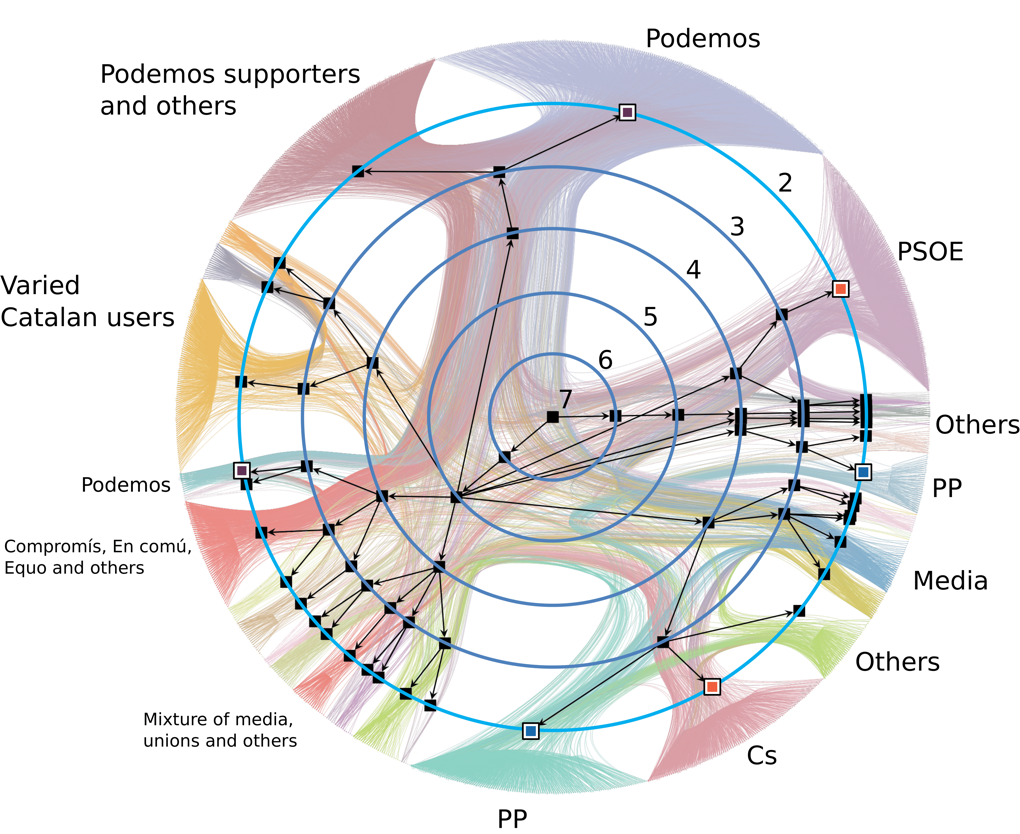


Note: The level used to build the elite is highlighted in light blue and the specific communities assigned to each pole are marked with squares of different colors. Blue: PP. Red: PSOE. Purple: Podemos. Orange: Cs.

**Figure 3A. Levels 2 to 7 of the hierarchical community structure for influential and engaged users of the 28A elections system.**


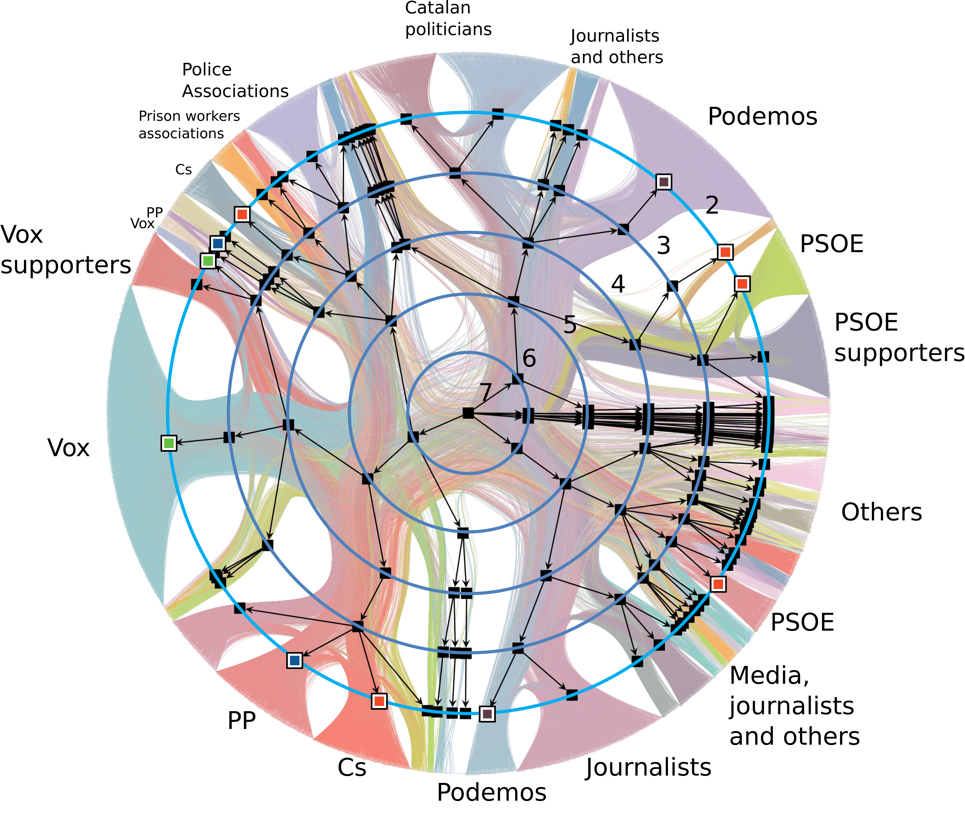


Note: The level used to build the elite is highlighted in light blue and the specific communities assigned to each pole are marked with squares of different colors. Blue: PP. Red: PSOE. Purple: Podemos. Orange: Cs. Green: Vox.

**Figure 4A. Levels 1 to 8 of the hierarchical community structure for influential and engaged users of the 10N elections system.**


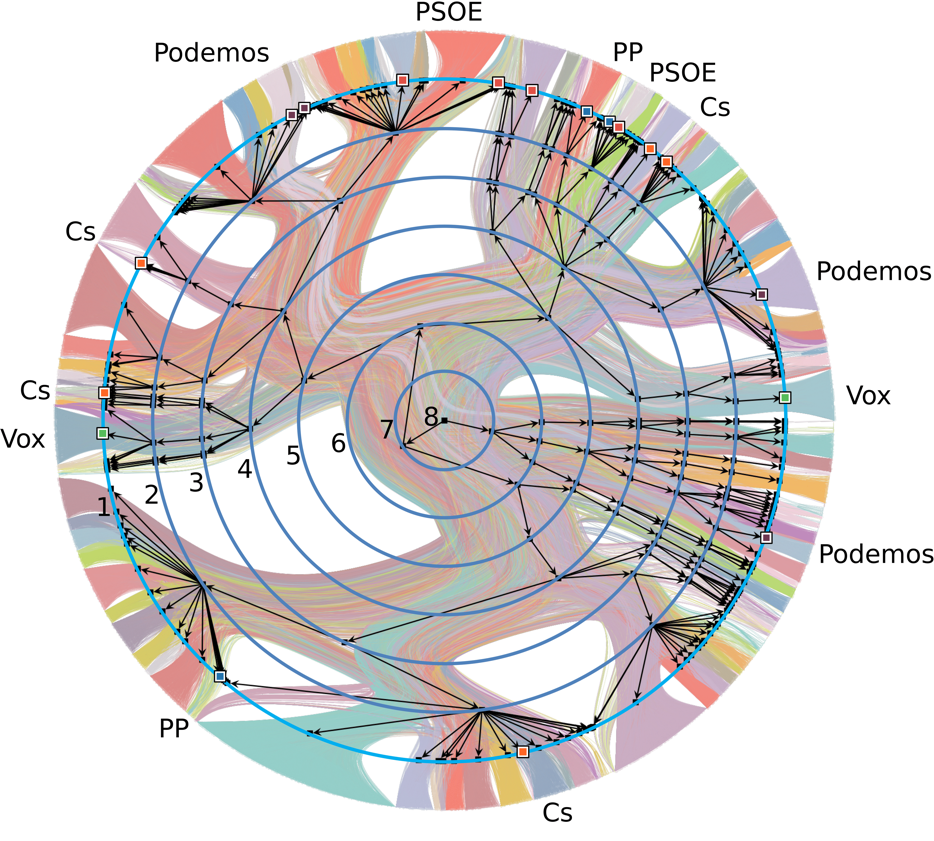


Note: The level used to build the elite is highlighted in light blue and the specific communities assigned to each pole are marked with squares of different colors. Blue: PP. Red: PSOE. Purple: Podemos. Orange: Cs. Green: Vox.

*3A. Sampling users from high relative user density areas and estimating their opinions*

To characterize the high relative user density areas in the opinion-efficiency maps, we have estimated the support for the different parties of the users whose opinion and efficiency values lie within them. To further focus the analysis, we have sampled users only from sub-regions with relative user density $P_{diff}\geq\alpha$, where $\alpha$ is a threshold that we have set to $\alpha=0.2$ for the 2015 and 2016 elections and to $\alpha=0.3$ for the two 2019 elections. In Table 3A, we detail the *total* number of users found in the high relative user density regions, the number of *filtered* users once we apply the $\alpha$ threshold, and the number of users we have *selected* to estimate party support. In total, we have selected 1947 users. These users were manually labeled by two annotators after reading the tweets they posted or retweeted in the corresponding conversation.

**Table 3A. Sampled users to estimate party support.**

|  | 2015 | | 2016 | | 2019 28A | | | 2019 10N | | | | |
| --- | --- | --- | --- | --- | --- | --- | --- | --- | --- | --- | --- | --- |
|  | A | B | A | B | A | B | D | A | B | C | D |  |
| Total | 1582 | 2368 | 2645 | 2358 | 7796 | 2690 | 26190 | 3622 | 4038 | 21652 | 24715 |  |
| Filtered | 867 | 975 | 1130 | 837 | 3321 | 1401 | 5196 | 2394 | 1876 | 491 | 6862 |  |
| Selected | 173 | 303 | 211 | 246 | 173 | 160 | 173 | 137 | 130 | 99 | 142 |  |

Note: Total number of users within each area of interest of the opinion-efficiency map, number of users in the high relative user density sub-regions of each area (Filtered), and number of randomly sampled users from those sub- regions (Selected).

Each user was labeled as a supporter or detractor of one or several of the main political parties according to the following criteria:

The classification is based only on explicit words, expressions, mentions, or hyperlinks, avoiding inferences, speculations, or assumptions.

- If there is reasonable evidence (words, mentions, etc.) to consider that a user supports a party, she will be labeled as a supporter of that party.
- If there is reasonable evidence (words, mentions, etc.) to consider that a user opposes a party, she will be labeled as a detractor of that party.
- If there is reasonable evidence (words, mentions, etc.) to consider that a user supports party A and opposes party B, she will be labeled as a supporter of A and detractor of B.
- If there is reasonable evidence (words, mentions, etc.) to consider that a user supports and/or opposes more than one party, she will be labeled as supporter/detractor of all the corresponding parties.
- If there is no clear evidence for declaring the user's support or opposition to any party, or the evidence is ambiguous, she is labeled as “unknown”.

Since we have focused on 5 different parties, each user can be theoretically assigned between 1 and 11 different labels: 5 for support, 5 for opposition, and one for “unknown”. Of course, there are incompatible combinations such as “unknown” with any of the other labels, or supporting and opposing a given party at the same time.

We tested these criteria by extracting a random sample of 60 users and measuring the inter-annotator agreement between the two annotators that worked on the project. For 93% of the users, they agreed in one or more labels.

**References:**

[1] J. Borondo, A. J. Morales, J. C. Losada, and R. M. Benito, ‘Characterizing and modeling an electoral campaign in the context of Twitter: 2011 Spanish Presidential election as a case study’, *Chaos Interdiscip. J. Nonlinear Sci.*, vol. 22, no. 2, p. 023138, Jun. 2012, doi: 10.1063/1.4729139.

[2] P. Barberá, J. T. Jost, J. Nagler, J. A. Tucker, and R. Bonneau, ‘Tweeting From Left to Right: Is Online Political Communication More Than an Echo Chamber?’, *Psychol. Sci.*, vol. 26, no. 10, pp. 1531–1542, Oct. 2015, doi: 10.1177/0956797615594620.

[3] P. Metaxas, E. Mustafaraj, K. Wong, L. Zeng, M. O’Keefe, and S. Finn, ‘What Do Retweets Indicate? Results from User Survey and Meta-Review of Research’, *Proc. Int. AAAI Conf. Web Soc. Media*, vol. 9, no. 1, Art. no. 1, 2015.

[4] M. H. Degroot, ‘Reaching a Consensus’, *J. Am. Stat. Assoc.*, vol. 69, no. 345, pp. 118–121, Mar. 1974, doi: 10.1080/01621459.1974.10480137.

[5] N. E. Friedkin and E. C. Johnsen, ‘Social influence networks and opinion change’, *Adv. Group Process.*, vol. 16, pp. 1–29, 1999.

[6] S. E. Parsegov, A. V. Proskurnikov, R. Tempo, and N. E. Friedkin, ‘Novel Multidimensional Models of Opinion Dynamics in Social Networks’, *IEEE Trans. Autom. Control*, vol. 62, no. 5, pp. 2270–2285, May 2017, doi: 10.1109/TAC.2016.2613905.

[7] A. J. Morales, J. Borondo, J. C. Losada, and R. M. Benito, ‘Measuring political polarization: Twitter shows the two sides of Venezuela’, *Chaos Interdiscip. J. Nonlinear Sci.*, vol. 25, no. 3, p. 033114, Mar. 2015, doi: 10.1063/1.4913758.

[8] J. Atienza-Barthelemy, S. Martin-Gutierrez, J. C. Losada, and R. M. Benito, ‘Relationship between ideology and language in the Catalan independence context’, *Sci. Rep.*, vol. 9, no. 1, Art. no. 1, Nov. 2019, doi: 10.1038/s41598-019-53404-x.

[9] S. Martin-Gutierrez, J. C. Losada, and R. M. Benito, ‘Multipolar social systems: Measuring polarization beyond dichotomous contexts’, *Chaos Solitons Fractals*, vol. 169, p. 113244, Apr. 2023, doi: 10.1016/j.chaos.2023.113244.

[10] T. P. Peixoto, ‘Hierarchical Block Structures and High-Resolution Model Selection in Large Networks’, *Phys. Rev. X*, vol. 4, no. 1, p. 011047, Mar. 2014, doi: 10.1103/PhysRevX.4.011047.
